# Analysis of *Torquetenovirus* DNA levels in NSCLC patients treated with immune checkpoint inhibitors

**DOI:** 10.1371/journal.pone.0349500

**Published:** 2026-05-15

**Authors:** Lilia Cinti, Lucrezia Tuosto, Piergiorgio Roberto, Roberta Campagna, Aurelia Rughetti, Chiara Napoletano, Alain J. Gelibter, Daniele Santini, Guido Antonelli, Ombretta Turriziani

**Affiliations:** 1 Department of Molecular Medicine, Laboratory of Microbiology and Virology, Sapienza University o f Rome, Rome, Italy; 2 Department of Medical Biotechnologies, PhD of National Interest in Innovation in the Diagnosis, Prevention and Treatment of Infections at Epidemic-Pandemic Risk, University of Siena, Siena, Italy; 3 University Hospital “Policlinico Umberto I”, Rome, Italy; 4 Department of Experimental Medicine, Laboratory of Tumor Immunology and Cell Therapies, Sapienza University of Rome, Rome, Italy; 5 Phd of Network Oncology and Precision Medicine, Sapienza University of Rome, Rome, Italy; 6 Department of Public Health, PhD National Programme in One Health approaches to Infectious Diseases and Life Science Research, Experimental and Forensic Medicine, University of Pavia, Pavia, Italy; 7 Division of Oncology, Department of Radiological, Oncological and Pathological Science, Sapienza University of Rome, Rome, Italy; 8 Department of Medico-Surgical Sciences and Biotechnologies, Sapienza University of Rome, Rome, Italy; Shanghai Public Health Clinical Center, Fudan University, CHINA

## Abstract

In recent years, *Torquetenovirus* (TTV) load has emerged as a potential biomarker of immune competence across various clinical contexts, albeit its significance in cancer patients undergoing immunotherapy remains largely unexplored. This study aimed to investigate the level of TTV viral load in non-small cell lung cancer (NSCLC) patients treated with immune checkpoint inhibitors (ICIs). A total of 64 NSCLC patients were enrolled. Serum TTV DNA levels were quantified by real-time PCR at baseline (T0) and after three months of ICI therapy (T1). As control, TTV levels were also measured in a cohort of healthy donors at a single time point. Statistical analyses were performed to compare TTV copy numbers across groups and time points. At baseline, cancer patients exhibited significantly higher TTV viral loads than healthy donors, consistent with an immunocompromised status. Following ICI treatment, a significant decline in TTV levels was observed in the oncologic cohort, reaching values comparable to those of healthy individuals, with a more pronounced decrease observed in patients responsive to therapy. This study provides the first evidence that TTV viral load decreases following immunotherapy in NSCLC patients, potentially reflecting a restoration of immune competence. These findings support the hypothesis that TTV monitoring could serve as a surrogate marker of immune function during ICI treatment. Further large-scale, longitudinal studies are warranted to validate its clinical meaning in oncologic settings.

## Introduction

*Torquetenovirus* (TTV) is a human virus belonging to the *Anelloviridae* family, with no established pathogenicity, whose viral load fluctuates in relation to the host’s immune function status [1]. Over the past years, TTV has gained increasing attention as a potential biomarker of immune competence across various clinical settings [2,3]. Notably, a number of studies involving solid organ transplant recipients and patients with chronic infections, such as HIV, have demonstrated that TTV viral load increases in conditions of immunosuppression. This leads to the suitable concept that TTV viral load may represent a sensitive indicator of the degree of immune dysfunction (for a recent review see Medina et al., 2025 [4]).

The role of TTV viral load as a potential immune biomarker in cancer patients undergoing immunotherapy remains largely unexplored. Specifically, systematic studies investigating the kinetics of TTV viral load in relation to immune modulation induced by immune checkpoint inhibitors (ICIs) in oncologic settings are currently absent. It still needs to be established whether changes in TTV levels can reliably reflect alterations in immune function occurring during immunotherapeutic treatment. It is tempting to speculate that this goal may be particularly interesting, also from a clinical perspective, since ICI therapies such as programmed cell death protein 1 (PD-1) axis blockade used either as monotherapy or in combination with conventional treatments such as chemotherapy, target therapies, and radiotherapy have achieved substantial success in the treatment of several types of cancers [5]. Indeed, it is increasingly recognized that not all patients benefit from ICI therapy, and clinical responses remain highly heterogeneous. This highlights the need for reliable predictive biomarkers (non-immune or immune based) capable of monitoring the patient’s immune status and identifying those more likely to respond effectively to immunotherapy [6].

This study retrospectively presents a provisional attempt to address the above issue. Specifically, TTV viral load was assessed in non-small cell lung cancer (NSCLC) patients treated with immunotherapy, compared to that of healthy individuals, and monitored throughout ICIs treatment.

## Materials and methods

This retrospective study was conducted on a total of 64 patients with metastatic non-oncogene-addicted NSCLC undergoing immunotherapy (anti-PD-1 antibody alone or in combination with anti-CTLA-4 and chemotherapy based on the tumor expression of PD-L1 levels) at the University Hospital Policlinico Umberto I. The median age was 70 years (IQR: 63–79), and 70% were male (Table 1). Serum samples were collected at diagnosis, before treatment initiation (T0, n = 64), and at the first clinical re-evaluation, 3 months after starting immunotherapy (T1, n = 49) (S1 Table), and were cryopreserved until use [7]. All patients included in the study had prednisone doses below 10 mg prior to initiation of immunotherapy and were not receiving any antiviral or other immunomodulatory medications at the time of sampling. A control group of 18 healthy donors (median age 53 years, IQR: 45–62) was also analyzed.

**Table 1:** Baseline demographic and treatment characteristics of patients with NSCLC receiving immune checkpoint inhibitor-based therapy and healthy controls.

Characteristic	Patients (n = 64)	Controls (n = 18)
**Age, median (IQR), years**	70 (63-79)	53 (45-62)
**Sex, n (%)**		
- Male	45 (70%)	9 (50%)
- Female	19 (30%)	9 (50%)
**Treatment type, n (%)**		–
- ICI monotherapy (Pembrolizumab)	22 (34%)	–
- ICI + chemotherapy (Pembrolizumab + chemotherapy)	20 (32%)	–
- ICI + chemotherapy + other immuno (Nivolumab + Ipilimumab + chemotherapy)	22 (34%)	–

The study was approved by the Territorial Ethics Committee Lazio Area 1 (CE protocol number: 0365/2025). Given the retrospective nature of the study, data were accessed for research purposes between 16/04/2025 and 15/05/2025. Informed consent was obtained from all participants.

TTV DNA quantification and amplification. Total nucleic acids were extracted from 300 µl of serum using the automated NucliSens easyMAG platform (bioMérieux, Marcy l’Etoile, France), in accordance with the manufacturer’s guidelines. Quantitative detection of *Torquetenovirus* (TTV) DNA was performed using the CFX96 real-time PCR detection system (Bio-Rad Laboratories, Hercules, CA, USA). The amplification protocol employed a set of primers and thermal cycling conditions specifically optimized for TTV genome quantification, as previously described by Roberto et al., 2023 [8].

Statistical analyses were performed using the Wilcoxon signed-rank test for paired data and the Mann-Whitney U test for unpaired data. Exact *p*-values were reported, along with the median of paired differences and their 95% confidence intervals where applicable. All tests were two-sided, and *p* < 0.05 was considered statistically significant.

## Results

In this study, 64 cancer patients were analyzed. TTV DNA levels were measured in serum samples collected at two different time points from NSCLC patients (T0 and T1), and at a single time point (T0) for healthy donors.

When stratifying the oncologic cohort, no significant differences in TTV viral load were observed across treatment regimens (monotherapy vs. combination therapy).

**Fig 1:**
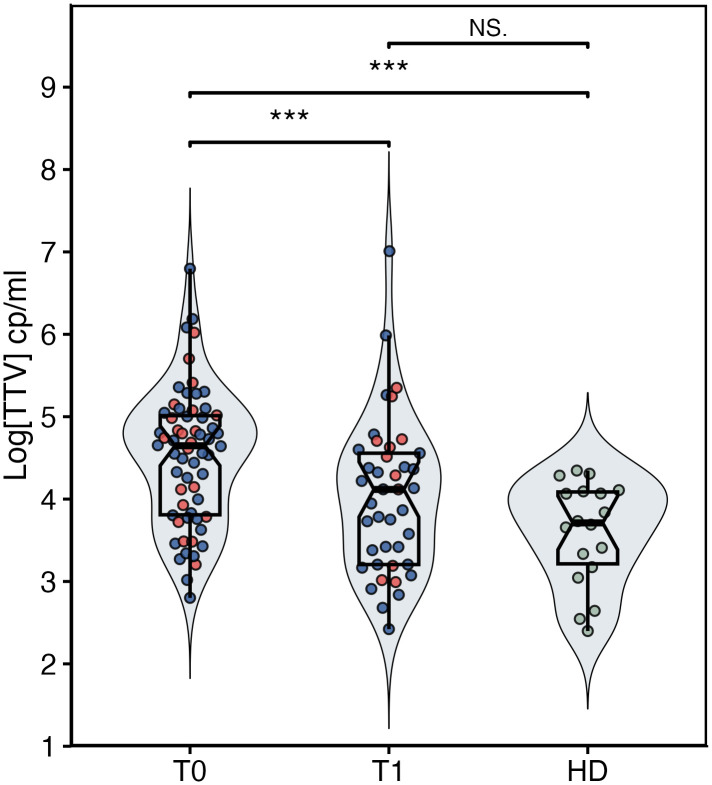
Comparison of TTV viral load in healthy donors (HD) and NSCLC patients undergoing immunotherapy at baseline (T0) and after treatment initiation (T1). Patients responsive to therapy are indicated by blue dots, whereas patients non-responsive to therapy are indicated by red dots. Statistical significance is indicated as follows: *p* < 0.05 (*), *p* < 0.01 (**), *p* < 0.001 (***).

Conversely, as displayed in Fig 1, cancer patients at T0 exhibited significantly higher TTV viral loads (median [IQR], 42,397 [6,045–101,392] copies/ml) compared to healthy donors (5,146 [1,659–12,211] copies/ml; *p* < 0.0001). Within the oncologic cohort, a significant decrease in TTV copy number was observed between T0 and T1 (5,650 [983–24,458] copies/ml; *p* = 0.0003) (Fig 1). No significant differences were observed between the TTV levels of healthy donors and cancer patients at T1 (Fig 1).

**Fig 2:**
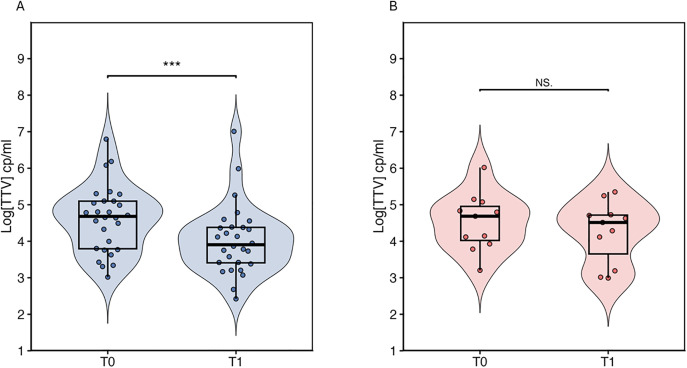
Comparison of TTV viral load in responders (A) and non-responders (B) among NSCLC patients undergoing immunotherapy at baseline (T0) and after treatment initiation (T1). Statistical significance is indicated as follows: *p* < 0.05 (*), *p* < 0.01 (**), *p* < 0.001 (***).

When stratifying patients according to treatment response at 3 months, responders (R) showed a significant reduction in TTV viral load between T0 (median [IQR], 35,546 [5,823–98,931]) and T1 (5,512 [1,256–22,590] copies/ml; *p* < 0.001). In contrast, non-responders (NR) did not exhibit a significant change in TTV levels over time (median [IQR], 55,283 [8,463–103,718] vs. 16,236 [492–46,738] copies/ml; *p* = ns) (Fig 2).

## Discussion

In patients with NSCLC, the identification of reliable biomarkers capable of predicting response to immunotherapy and monitoring immune status remains a major clinical challenge [9]. Although expression of tumor PD-L1 is currently the most widely used biomarker to guide therapeutic decisions, it does not fully reflect the patient’s immune competence, particularly in complex and dynamic oncological settings [9].

In this framework, our study might provide the first evidence that cancer immunotherapy induces measurable changes in TTV viral load in NSCLC patients. At diagnosis, cancer patients displayed markedly higher TTV levels compared with healthy subjects, consistent with an underlying immune dysfunction. After three months of ICI treatment, TTV levels decreased significantly and reached values comparable to healthy controls, suggesting a modulation or partial restoration of immune competence. Notably, this reduction in TTV viral load appeared to be driven by patients who responded to therapy, in whom a significant decline was observed over time. In contrast, non-responders did not exhibit a comparable decrease in TTV levels, suggesting a lack of effective immune restoration in this subgroup. These findings support the hypothesis that TTV viral load may reflect changes in immune status induced by the immunotherapy.

Age-related differences between patients and controls may partly influence TTV variability [10], but the convergence of TTV levels after treatment strongly suggests that immune modulation by ICIs is the main driver of this effect.

Interestingly, these findings raise the hypothesis that immunotherapy-mediated immune reconstitution may enhance the host’s capacity to control TTV replication. In this perspective, monitoring TTV dynamics during ICIs could not only provide a surrogate marker of immune competence but also contribute to a better understanding of the mechanisms underlying the immunological control of anelloviruses.

However, since a proportion of these patients undergo combination therapy with ICIs and chemotherapy, it cannot be excluded that the observed results may be influenced, at least in part, by the effects of chemotherapy.

Due to the limited number of patients included, the study was designed just as a proof-of-concept to evaluate the potential use of TTV as an immune marker in oncologic patients undergoing immunotherapy. These preliminary results encourage further studies to explore this approach more extensively.

## Supporting information

S1 Table Demographic and clinical characteristics of the study cohort. **The table reports anonymized participant ID, sex, age, therapy responsiveness and treatment regimen for oncological patients and healthy donors.** (O, oncological patient; HD, healthy donor; F, female; M, male; R, responder; NR, non-responder; N/A, not applicable.).
(DOCX)
