# Supplementary material for: Analysis of Torquetenovirus DNA levels in NSCLC patients treated with immune checkpoint inhibitors
Source: PLoS One. 2026 May 15;21(5):e0349500. doi: 10.1371/journal.pone.0349500 (PMC13178919; doi:10.1371/journal.pone.0349500)
Supplement: S1 Table — The table reports anonymized participant ID, sex, age, therapy responsiveness and treatment regimen for oncological patients and healthy donors. (O, oncological patient; HD, healthy donor; F, female; M, male; R, responder; NR, non-responder; N/A, not applicable.). (DOCX) [file pone.0349500.s001.docx]

**Table S1.**

| **ID** | **Sex** | **Age** | **Therapy Responsiveness** | **Therapy** |
| --- | --- | --- | --- | --- |
| 1-O | F | 82 | R | Pembrolizumab |
| 2-O | M | 77 | NR | Pembrolizumab |
| 3-O | M | 81 | R | Pembrolizumab |
| 4-O | M | 52 | NR | Pembrolizumab |
| 5-O | M | 63 | NR | Pembrolizumab |
| 6-O | F | 67 | R | Pembrolizumab |
| 7-O | F | 79 | R | Pembrolizumab |
| 8-O | M | 60 | NR | Pembrolizumab |
| 9-O | M | 82 | R | Pembrolizumab |
| 10-O | M | 71 | NR | Pembrolizumab |
| 11-O | M | 92 | NR | Pembrolizumab |
| 12-O | M | 61 | NR | Pembrolizumab |
| 13-O | M | 88 | R | Pembrolizumab |
| 14-O | F | 75 | R | Pembrolizumab |
| 15-O | M | 60 | R | Pembrolizumab |
| 16-O | M | 66 | NR | Pembrolizumab |
| 17-O | M | 79 | NR | Pembrolizumab |
| 18-O | M | 80 | R | Pembrolizumab |
| 19-O | M | 52 | R | Pembrolizumab |
| 20-O | F | 89 | R | Pembrolizumab |
| 21-O | M | 68 | R | Pembrolizumab |
| 22-O | M | 68 | R | Pembrolizumab |
| 23-O | F | 67 | R | Pembrolizumab + chemotherapy |
| 24-O | M | 68 | R | Pembrolizumab + chemotherapy |
| 25-O | F | 77 | NR | Pembrolizumab + chemotherapy |
| 26-O | M | 80 | R | Pembrolizumab + chemotherapy |
| 27-O | M | 63 | R | Pembrolizumab + chemotherapy |
| 28-O | F | 61 | NR | Pembrolizumab + chemotherapy |
| 29-O | M | 63 | R | Pembrolizumab + chemotherapy |
| 30-O | F | 68 | R | Pembrolizumab + chemotherapy |
| 31-O | F | 71 | R | Pembrolizumab + chemotherapy |
| 32-O | F | 76 | R | Pembrolizumab + chemotherapy |
| 33-O | M | 77 | NR | Pembrolizumab + chemotherapy |
| 34-O | M | 74 | NR | Pembrolizumab + chemotherapy |
| 35-O | M | 90 | NR | Pembrolizumab + chemotherapy |
| 36-O | M | 63 | R | Pembrolizumab + chemotherapy |
| 37-O | M | 50 | R | Pembrolizumab + chemotherapy |
| 38-O | F | 79 | R | Pembrolizumab + chemotherapy |
| 39-O | M | 52 | R | Pembrolizumab + chemotherapy |
| 40-O | M | 84 | R | Pembrolizumab + chemotherapy |
| 41-O | M | 63 | NR | Pembrolizumab + chemotherapy |
| 42-O | F | 73 | R | Pembrolizumab + chemotherapy |
| 43-O | M | 56 | R | Nivolumab + Ipilimumab + chemotherapy |
| 44-O | M | 74 | NR | Nivolumab + Ipilimumab + chemotherapy |
| 45-O | M | 71 | NR | Nivolumab + Ipilimumab + chemotherapy |
| 46-O | M | 60 | R | Nivolumab + Ipilimumab + chemotherapy |
| 47-O | M | 80 | R | Nivolumab + Ipilimumab + chemotherapy |
| 48-O | M | 63 | R | Nivolumab + Ipilimumab + chemotherapy |
| 49-O | M | 68 | R | Nivolumab + Ipilimumab + chemotherapy |
| 50-O | M | 74 | R | Nivolumab + Ipilimumab + chemotherapy |
| 51-O | M | 64 | NR | Nivolumab + Ipilimumab + chemotherapy |
| 52-O | F | 80 | R | Nivolumab + Ipilimumab + chemotherapy |
| 53-O | F | 70 | R | Nivolumab + Ipilimumab + chemotherapy |
| 54-O | F | 89 | R | Nivolumab + Ipilimumab + chemotherapy |
| 55-O | M | 60 | R | Nivolumab + Ipilimumab + chemotherapy |
| 56-O | M | 67 | R | Nivolumab + Ipilimumab + chemotherapy |
| 57-O | M | 66 | R | Nivolumab + Ipilimumab + chemotherapy |
| 58-O | F | 64 | NR | Nivolumab + Ipilimumab + chemotherapy |
| 59-O | F | 76 | R | Nivolumab + Ipilimumab + chemotherapy |
| 60-O | M | 72 | R | Nivolumab + Ipilimumab + chemotherapy |
| 61-O | M | 83 | NR | Nivolumab + Ipilimumab + chemotherapy |
| 62-O | M | 77 | R | Nivolumab + Ipilimumab + chemotherapy |
| 63-O | F | 61 | NR | Nivolumab + Ipilimumab + chemotherapy |
| 64-O | M | 56 | R | Nivolumab + Ipilimumab + chemotherapy |
| 1-HD | M | 50 | N/A | N/A |
| 2-HD | F | 25 | N/A | N/A |
| 3-HD | M | 63 | N/A | N/A |
| 4-HD | M | 53 | N/A | N/A |
| 5-HD | M | 57 | N/A | N/A |
| 6-HD | F | 43 | N/A | N/A |
| 7-HD | M | 51 | N/A | N/A |
| 8-HD | M | 51 | N/A | N/A |
| 9-HD | M | 27 | N/A | N/A |
| 10-HD | F | 26 | N/A | N/A |
| 11-HD | F | 59 | N/A | N/A |
| 12-HD | F | 50 | N/A | N/A |
| 13-HD | F | 29 | N/A | N/A |
| 14-HD | F | 60 | N/A | N/A |
| 15-HD | F | 64 | N/A | N/A |
| 16-HD | M | 66 | N/A | N/A |
| 17-HD | M | 67 | N/A | N/A |
| 18-HD | F | 63 | N/A | N/A |

O, oncological patient; HD, healthy donor; F, female; M, male; R, responder; NR, non-responder; N/A, not applicable.
